# Characterization and Analysis of the Mitochondrial Genome of Common Bean (*Phaseolus vulgaris*) by Comparative Genomic Approaches

**DOI:** 10.3390/ijms21113778

**Published:** 2020-05-27

**Authors:** Changwei Bi, Na Lu, Yiqing Xu, Chunpeng He, Zuhong Lu

**Affiliations:** 1 State Key Laboratory of Bioelectronics, School of Biological Science and Medical Engineering, Southeast University, Nanjing 210096, Jiangsu, China; bichwei@seu.edu.cn (C.B.); nlu@seu.edu.cn (N.L.); 2 School of Information Science and Technology, Nanjing Forestry University, Nanjing 210037, Jiangsu, China; yiqingxu@njfu.edu.cn

**Keywords:** common bean, *Phaseolus vulgaris*, mitochondrial genome, comparative genomics, phylogeny

## Abstract

The common bean (*Phaseolus vulgaris*) is a major source of protein and essential nutrients for humans. To explore the genetic diversity and phylogenetic relationships of *P. vulgaris*, its complete mitochondrial genome (mitogenome) was sequenced and assembled. The mitogenome is 395,516 bp in length, including 31 unique protein-coding genes (PCGs), 15 transfer RNA (tRNA) genes, and 3 ribosomal RNA (rRNA) genes. Among the 31 PCGs, four genes (*mttB*, *nad1*, *nad4L*, and *rps10*) use ACG as initiation codons, which are altered to standard initiation codons by RNA editing. In addition, the termination codon CGA in the *ccmF_C_* gene is converted to UGA. Selective pressure analysis indicates that the *ccmB*, *ccmF_C_*, *rps1*, *rps10*, and *rps14* genes were under evolutionary positive selection. The proportions of five amino acids (Phe, Leu, Pro, Arg, and Ser) in the whole amino acid profile of the proteins in each mitogenome can be used to distinguish angiosperms from gymnosperms. Phylogenetic analyses show that *P. vulgaris* is evolutionarily closer to the Glycininae than other leguminous plants. The results of the present study not only provide an important opportunity to conduct further genomic breeding studies in the common bean, they also provide valuable information for future evolutionary and molecular studies of leguminous plants.

## 1. Introduction

Mitochondria (mt) are semi-autonomous organelles that are part of almost all eukaryotic cells (cells with clearly defined nuclei). Their primary function is to produce a steady supply of adenosine triphosphate (ATP). Mitochondria are thus termed the ‘powerhouses’ or ‘energy factories’ of cells. Chloroplasts (cp) and mitochondria most likely originated from formerly free-living bacteria through endosymbiotic acquisition, which can explain the presence of their own genomes [1,2]. With rapid developments in sequencing and genome assembly methods, an increasing number of complete organelle genomes have been assembled in the last decade. Thus far, over 4900 complete chloroplast and plastid genomes have been assembled but only 321 plant mitogenomes have been assembled and deposited in GenBank Organelle Genome Resources (as of 14 May 2020; https://www.ncbi.nlm.nih.gov/genome/browse/), suggesting that their assembly is complex and difficult.

Mitochondria are specific to each plant and have complex genome structures [3,4,5], variable genome sizes [6,7], numerous repetitive sequences [8,9], multiple RNA editing modifications [10,11], and frequent gene gains or losses during evolution [9,12,13]. In seed plant mitogenomes, the genome sizes are highly variable, ranging from an exceptionally small genome of 66 kb in the parasitic plant *Viscum scurruloideum* [14] to the largest multi-chromosomal genome of 11.3 Mb in *Silene conica* [15]. Even if two species are evolutionarily close, their genome sizes may vary considerably. The mitogenome sizes of plants in the subfamily Papilionoideae range from 271 kb in *Medicago truncatula* [16] to 588 kb in *Vicia faba* [17], while the mitogenomes of most papilionoid legumes are approximately 400 kb in length [18]. This wide variation in mitogenome size can be attributed to the proliferation of repetitive sequences and the acquisition of foreign DNA from other organisms during evolution [19,20].

Previous studies have documented that the mitogenomes of seed plants are enriched with repetitive sequences, including simple sequence repeats (SSRs), tandem repeats, and dispersed repeats. The SSRs in plant mitogenomes are commonly used as molecular markers for studying genetic diversity and identifying species [21]. The tandem repeats occur in a broad range of plant mitogenomes, which can also serve as molecular markers for unravelling population processes in plants [22]. Large dispersed repeats are the main causes of genome rearrangements, which may generate multipartite structures [13,23,24,25].

Although the mitogenome sizes of seed plants are variable, the functional genes of NADH dehydrogenase, ubiquinol cytochrome c reductase, ATP synthase, and cytochrome c biogenesis are quite conservative, except for succinate dehydrogenase genes and ribosomal proteins. Many primordial mt genes have been lost during evolution, which has been found to be closely related to their specific functions. For example, *sdh3* and *sdh4* were lost in all gramineous mitogenomes, the *rps11* gene was lost in the differentiation of gymnosperms and angiosperms [26], and the *cox2* gene was lost in the differentiation of the Phaseoleae and Glycininae [18]. Strikingly, nearly all of the universally present NADH dehydrogenase genes were lost from the mitogenome of *Viscum scurruloideum*, with the loss closely associated with its parasitic lifestyle [14].

The Fabaceae, commonly known as legumes, is an economically and ecologically important family of flowering plants ranging from small annual herbs to giant trees, most of which are herbaceous perennials. This family is the third-largest angiosperm family after the Asteraceae and Orchidaceae [27,28], consisting of about 770 genera and more than 20,000 species. A recent study by the Legume Phylogeny Working Group (LPWG) reclassified the three widely-accepted Fabaceae subfamilies (Caesalpinioideae, Minosoideae, and Papilionoideae) into six new subfamilies (Cercidoideae, Detarioideae, Duparquetioideae, Dialioideae, Caesalpinioideae, and Papilionoideae) based on a taxonomically-comprehensive phylogeny [28]. However, due to the complexity of plant mitogenomes, only 27 mitogenomes of Fabaceae species have been assembled and deposited in the NCBI Nucleotide database (14 May 2020), including 19 species in the Papilionoideae, six species in the Caesalpinioideae, one species of *Cercis canadensis* in the Cercidoideae, and one species of *Tamarindus indica* in the Detarioideae.

In this study, we assembled the complete mitogenome of the common bean *Phaseolus vulgaris*, an herbaceous annual plant grown worldwide for its edible dry seeds or unripe fruit. The common bean is one of the most important grain legumes for human consumption and plays an important role in sustainable agriculture due to its ability to fix atmospheric nitrogen [29]. We analyzed its gene content, repetitive sequences, RNA editing sites, selective pressure, and phylogenetic position, then made comparisons with other plant mitogenomes. The complete mitogenome of *P. vulgaris* will provide important information for the investigation of mitogenomic evolution among the Fabaceae family and aid the functional study of fabaceous mitogenomes. Mitochondrial biogenesis is very important in plant breeding and knowledge of the complete mitogenome provides an opportunity to conduct further important genomic breeding studies in the common bean.

## 2. Materials and Methods

### 2.1. Plant Materials, DNA Extraction, and Sequencing

An inbred landrace of *P. vulgaris* accession G19833 derived from the Andean pool (Race Peru) was selected for sequencing. Seeds were obtained from the germplasm bank of the Embrapa Arroz e Feijão, Brazil [30]. Root tips obtained from germinated seeds were pre-treated with 2 mM 8-hydroxyquinoline for 18 h at 10 °C, fixed in ethanol-acetic acid (3:1 *v*/*v*), and stored in fixative at −20 °C for up to several weeks. Total genomic DNA was extracted from root tips using DNAeasy Plant Mini Kits (Qiagen). To construct the shotgun library, DNA was fragmented by nebulization. The raw reads were sequenced with a combination of Roche/454 GS FLX sequencing reads, Illumina HiSeq-2500 sequencing short reads (primarily to correct 454 sequencing errors) and PacBio RS II sequencing long reads (primarily to validate the assembly of the master conformation). The raw reads of *P. vulgaris* used in this study were available in the NCBI Sequence Read Archive (SRA) under accessions SRR069592, SRR5628227, and SRR2912756.

### 2.2. Mitogenome Assembly and Annotation

**Figure 1:**
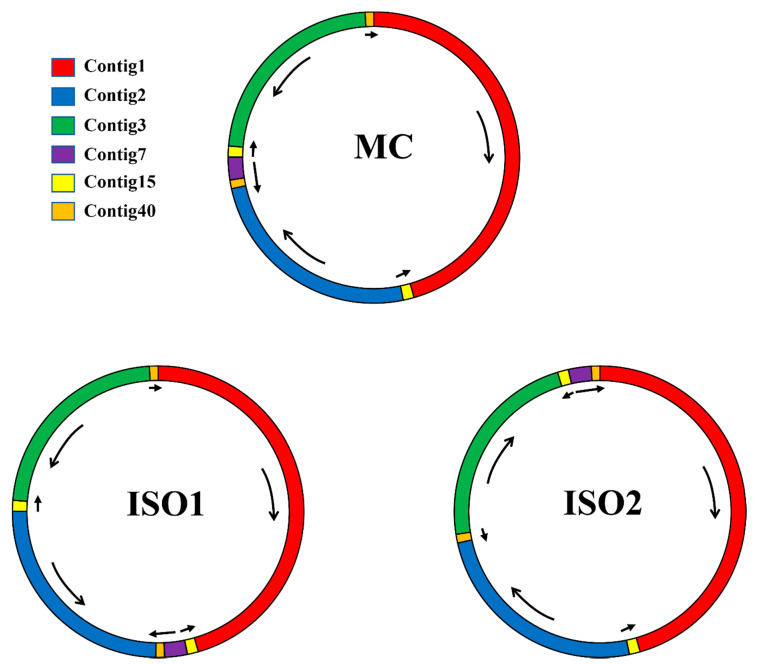
The master genome and two isomeric genomes observed from *P. vulgaris* mitogenome mediated by two pairs of large repeats (Contig15 and Contig40). The mt contigs were generated and selected from Newbler assembly software. MC and ISO mean the master and isomeric conformations, respectively. Arrows denote the sequence orientation of assembled contigs.

An efficient procedure for plant mitochondrial genome assembly using whole-genome data from the 454 GS FLX sequencing platform has been applied in many plants, such as *Boea hygrometrica* [31], *Daucus carota* [32], *Gossypium raimondii* [26], and *Salix suchowensis* [33]. Briefly, as shown in Figure S1, we first assembled all the Roche/454 GS FLX sequencing reads using Newbler (version 3.0) [34] with the following parameters: -cpu 20, -het, -sio, -m, -urt, -large, and -s 100. Then, we used custom Perl scripts to construct a draft assembly graph from the file “454AllContigGraph.txt” generated from Newbler. As shown in Figure 1, we obtained six contigs to construct the completed draft mitochondrial graph for assembling the *P. vulgaris* mitogenome. Among the six selected contigs, two (Contig15 and Contig40) were assembled into the mitogenome twice, while the others were assembled only once. To assemble the master conformation (MC), we mapped the PacBio sequencing reads to the mt contigs that spanned repetitive contigs using BLASTN to obtain a major contig relationship map for the repeat regions [35,36].

Specifically, for each repeat pair (Contig15 and Contig40), we built four reference sequences according to Dong et al. [37], each with 200 bp up- and down-stream of the two template sequences (original sequences). Then, we searched the PacBio long reads against the database built up from the reference sequences and extracted the matching reads with a blast identity above 80%, an e-value cut-off of 1e^−100^, and a hit length of over 3000 bp. Next, we mapped the best-matched reads to the four reference sequences in MacVector v17.0.7. As shown in Figure 1, we obtained one master genome and two isomeric genomes (ISO) based on the number of PacBio reads that were mapped to both end contigs of the repetitive contigs (Table S1). We then mapped Illumina sequencing reads to the draft MC mitogenome with BWA [38] and SAMtools [39] softwares to correct the homopolymer length errors (especially in A/T enriched regions) from 454 GS FLX Titanium [26]. Finally, the complete mitogenome sequence of *P. vulgaris* was obtained.

The mitogenome was annotated using the public MITOFY analysis web server (http://dogma.ccbb.utexas.edu/mitofy/) [8]. The putative genes were manually checked and adjusted by comparing them with other legume mitogenomes in MacVector v.17.07. All transfer RNA genes were confirmed by using tRNAscan-SE with default settings [40]. The start and stop codons of PCGs were manually adjusted to fit open reading frames. The relative synonymous codon usage (RSCU) values and amino acid composition of PCGs were calculated by MEGA X [41]. The OrganellarGenomeDRAW (OGDRAW) program was used to visualize the circular map of the *P. vulgaris* mitogenome [42].

### 2.3. Selective Pressure Analysis

To reflect the selective pressure of PCGs, we calculated the nonsynonymous (K_a_) and synonymous (K_s_) substitution rates of each PCG between *P. vulgaris* and other higher plants. *Arabidopsis thaliana* (*A. thaliana*; Brassicaceae) is a popular model organism in plant biology and genetics. *Citrullus lanatus* (*C. lanatus*; Cucurbitaceae) and *Vitis vinifera* (*V. vinifera*; Vitaceae) are highly cultivated fruits worldwide and belong to the Rosids clade, like leguminous plants. Therefore, we selected the mitogenomes of *A. thaliana*, *V. vinifera*, and *C. lanatus* as references to infer the direction and magnitude of natural selection acting on PCGs during the evolution of *P. vulgaris*. The orthologous gene pairs from *P. vulgaris*, *A. thaliana*, *V. vinifera*, and *C. lanatus* were aligned and formatted by ParaAT2.0 with default parameters [43]. The K_a_, K_s_, and K_a_/K_s_ values were calculated using KaKs_Calculator v.2.0 based on the YN method, and Fisher’s exact test was performed to justify the validity of the K_a_ and K_s_ values [44,45].

### 2.4. Prediction of RNA Editing Sites

The online PREP-Mt (predictive RNA editors for plants) suite of servers (http://prep.unl.edu/) was used to predict the possible RNA editing sites in the PCGs of *P. vulgaris* and the other four leguminous mitogenomes (*G. max*, *L. japonicus*, *V. radiata*, and *M. pinnata*). In order to predict more true RNA editing sites, the cut-off for prediction score was set as C = 0.2, which has been proven to be a slight optimum [46]. A low cut-off value will predict more true edit sites but will also increase the probability of misidentifying an unedited site as an edited one.

### 2.5. Identification of Repeat Sequences in P. vulgaris Mitogenome

Three kinds of repeats—SSRs, tandem repeats and dispersed repeats—were detected in the *P. vulgaris* mitogenome. The SSRs were detected using the web-based microsatellite identification tool MISA-web (https://webblast.ipk-gatersleben.de/misa/) [47] according to the methods of previous studies [9,26] with a motif size of one to six nucleotides and thresholds of eight, four, four, three, three, and three, respectively. Tandem repeats were identified using the online tool Tandem Repeats Finder 4.09 with default settings (http://tandem.bu.edu/trf/trf.html) [48]. AB-BLAST 3.0 was utilized to identify and locate dispersed repeats with the following parameters: M = 1, N = −3, Q = 3, R = 3, kap, span, B = 1 × 10^9^ and W = 7, which have been proven as effective in many studies [8,9,49]. The BLAST hits with e-values < 1 and identities > 80% were considered as disperse repeats. The number of dispersed repeats was calculated for seven size intervals (30–49, 50–69, 70–99, 100–149, 150–199, 200–999 and ≥1000 bp). Partly or wholly overlapping repeats were considered as a single repeat unit.

### 2.6. Phylogenetic Analyses

In order to accurately infer the phylogenetic relationships of *P. vulgaris* within the Fabaceae family, maximum likelihood (ML) analysis was performed based on the conserved mitochondrial PCGs (amino acid sequences) of 23 higher plants. The NCBI accession numbers and abbreviations of all these observed mitogenomes are listed in Table S2. Apart from the 19 representative Fabaceae species, taxon sampling also included two species of Solanales (*C. annuum* and *N. tabacum*) and two species of Malpighiales (*P. tremula* and *S. suchowensis*) as outgroups. The single-copy orthologous PCGs common among the 23 analysed species were selected with local Perl scripts. All conserved mitochondrial PCGs were extracted from each mitogenome. The conserved gene sequences from the mitogenome were concatenated into a single dataset and aligned using Muscle software with default settings [50]. Poorly-aligned sequences were deleted or manually adjusted for each alignment. Prior to constructing the phylogenetic tree, we applied MEGA X to determine the most appropriate amino acid substitution model [41]. Based on the model selection results, the ML tree based on a JTT + F model with a gamma distribution was constructed using MEGA X. The bootstrap index value (%) in which the associated taxa clustered together was shown next to the branches and was calculated from 1000 replications.

## 3. Results and Discussion

### 3.1. Genomic Features of the P. vulgaris Mitogenome

**Figure 2:**
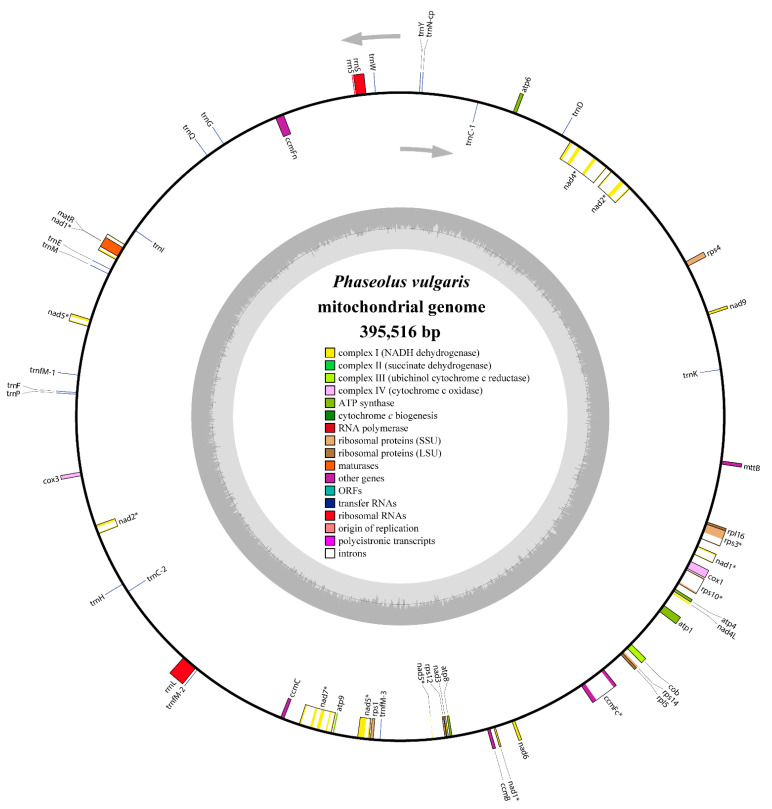
Circular map of the *P. vulgaris* mitogenome. Genes shown on the outside of the circle are transcribed clockwise, whereas genes on the inside are transcribed counterclockwise. GC content is represented on the inner circle by the dark gray plot. The asterisks besides genes denote intron-containing genes.

The complete genomic sequence of the *P. vulgaris* mitogenome was submitted to the NCBI Genome Database (https://www.ncbi.nlm.nih.gov/genome/browse/) under accession number NC_045135.1. The mitogenome was assembled into a typical circular molecule 395,516 bp in length (Figure 2), similar to the mitochondria of some papilionoid legumes such as *G. max* (402,558 bp), *G. soja* (402,545 bp), *L. japonicus* (380,861 bp), *V. angularis* (404,466 bp), and *V. radiata* var. *radiata* (401,262 bp; Table S2). In fact, the mitogenome sizes vary considerably among the papilionoid legumes, ranging from 271,618 bp in *Medicago truncatula* to 588,000 bp in *Vicia faba*. Mitogenome sizes can vary greatly in different cultivars of the same species. For example, the mitogenome size of *G. max* Aiganhuang (N21249) is 402,558 bp, whereas that of *G. max* cultivar Zhonghuang 13 is 513,779 bp [51].

The nucleotide composition of the whole mitogenome is A: 27.37%, C: 22.40%, G: 22.71%, and T: 27.52% (Table 1). The overall GC content is 45.11%, which is consistent with other leguminous plants (*G. max*: 45.03%, *V. faba*: 45.04%, and *V. radiata* var. *radiata*: 45.11%). Strikingly, the GC content of the PCGs is very small compared to those of other regions. As shown in Table 2, a total of 49 unique genes were detected in the *P. vulgaris* mitogenome, comprising 31 PCGs, 15 tRNA genes and 3 rRNA genes. However, none of the genes encodes subunits of Complex II (succinate dehydrogenase), which has also been lost in some other leguminous plants. Additionally, two tRNA genes located in repeat sequences were found to contain two or three copies (*trnC-GCA* and *trnfM-CAU*). The total lengths of the PCGs and *cis*-spliced introns comprise 7.26% and 8.24% of the whole mitogenome, while tRNA and rRNA genes only comprise 0.34% and 1.33% of the mitogenome, respectively. Most PCGs have no introns; however, eight genes (Table 2; *nad1*, *nad2*, *nad4*, *nad5*, *nad7*, *ccmF_C_*, *rps3*, and *rps10*) were found to contain one or more introns. Three genes (*nad1*, *nad2*, and *nad5*) required trans-splicing to assembly fully-translatable mRNA (Figure 2).

### 3.2. Codon Usage Analysis of PCGs

**Figure 3:**
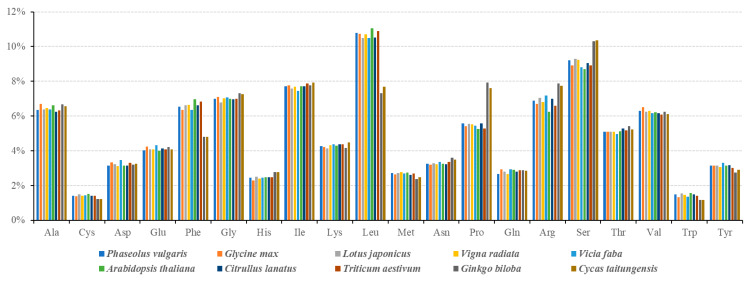
Codon usage pattern of *P. vulgaris* mitogenome compared with nine other higher plants. The proportion of each amino acid residues to the whole mitochondrial proteins is shown on the Y-axis. *Ginkgo biloba* and *Cycas taitungensis* are gymnosperms, while others are angiosperms.

In the *P. vulgaris* mitogenome, most of the PCGs use ATG as the start codon, while *mttB* and *nad1* start with ACG (C to U RNA editing on the second site is presumed) as the start codon (Table 3). Four types of stop codons were found in the PCGs: (1) TAA (15 genes; *atp4*, *atp8*, *atp9*, *cox1*, *nad1*, *nad2*, *nad3*, *nad4L*, *nad5*, *nad6*, *nad9*, *rpl5*, *rpl16*, *rps1*, and *rps4*), (2) TGA (10 genes; *atp1*, *ccmB*, *ccmC*, *ccmF_N_*, *cox3*, *matR*, *mttB*, *nad4*, *rps10*, and *rps12*), (3) TAG (5 genes; *atp6*, *cob*, *nad7*, *rps3*, and *rps14*), and (4) CGA (*ccmF_C_*; C to U RNA editing on the first site is presumed). As shown in Figure 3, the codon usage analysis revealed that leucine (Leu) and serine (Ser) are the most frequently-used amino acid residues, while cysteine (Cys) and tryptophan (Trp) are the least-used amino acid residues in the plant mitochondrial proteins. By comparison of the composition of *P. vulgaris* with other angiosperms plants, we found that the distribution of amino acid residues across the mitochondrial proteins are very similar in angiosperms (Figure 3). In addition, most of the amino acid residues were found to be very conserved between angiosperms (*P. vulgaris*, *G. max*, *L. japonicus*, *V. radiata*, *V. faba*, *A. thaliana*, *C. lanatus*, and *T. aestivum*) and gymnosperms (*Ginkgo biloba* and *Cycas taitungensis*), except for five of them (Phe, Leu, Pro, Arg, and Ser).

**Figure 4:**
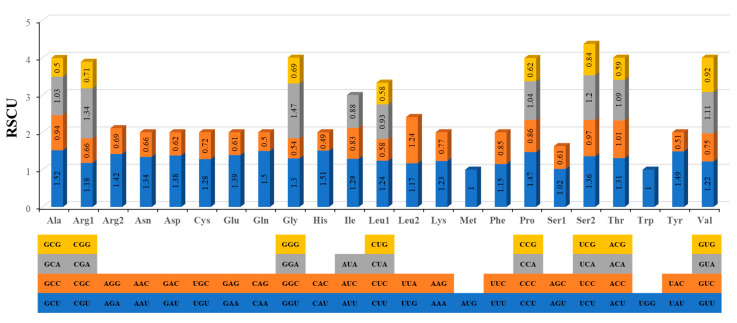
Relative synonymous codon usage (RSCU) of *P. vulgaris* mitogenome. Codon families are on the X-axis. RSCU values are the number of times of a particular codon, relative to the number of times that the codon would be observed for a uniform synonymous codon usage.

The relative synonymous codon usage (RSCU) analysis for the *P. vulgaris* mitogenome is shown in Figure 4, which indicates that all codons are present in the PCGs. Excluding the termination codons, the 31 PCGs in the *P. vulgaris* mitogenome consist of 9545 codons in total. Additionally, the codon usage showed that the RSCU values of the NNT and NNA codons are higher than 1.0 except for Ile (AUA) and Leu (CUA; Figure 4), suggesting a strong As or Ts bias in the third codon position of *P. vulgaris* mitochondrial PCGs, which is a very common phenomenon observed in all studied mitogenomes (Table S3). The codon usage pattern of *P. vulgaris* mitogenome is highly consistent with two other papilionoid legumes. The distributions of some codons encoding Pro (CCU, CCA, and CCG) differ between dicotyledons (*P. vulgaris*, *G. max*, *V. angularis*, *C. lanatus*, and *A. thaliana*) and monocotyledons (*T. aestivum*), and some codons (UCG, AGU, AGC, CCU, CCG, ACG, CGG, and AGA) are distributed differently between angiosperms and gymnosperms.

### 3.3. Selective Pressure Analysis

In genetics, the K_a_/K_s_ ratio is useful for inferring the direction and magnitude of natural selection acting on homologous PCGs across diverged species. The ratio is a more powerful test of the neutral model of evolution than many others available in population genetics as it requires fewer assumptions [52]. A K_a_/K_s_ ratio <1 implies purifying or stabilizing selection (acting against change), while a ratio of >1 implies positive or Darwinian selection (driving change) and a ratio of exactly 1 indicates neutral selection. Importantly, the K_a_/K_s_ ratio is unlikely to be significantly above 1 without at least some of the mutations being advantageous.

**Figure 5:**
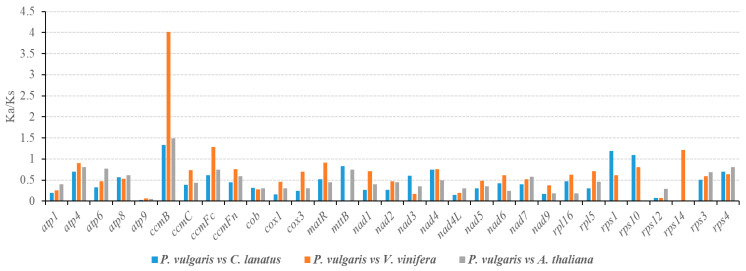
K_a_/K_s_ ratios for 31 protein coding genes of *P. vulgaris*, *C. lanatus*, *V. vinifera*, and *A. thaliana*. The blue, orange, and gray boxes indicate K_a_/K_s_ ratios of *P. vulgaris* vs. *C. lanatus*, *P. vulgaris* vs. *V. vinifera*, and *P. vulgaris* vs. *A. thaliana*.

In this study, the K_a_/K_s_ ratio was determined for all 31 PCGs following comparison of the *P. vulgaris* mitogenome with those of *C. lanatus*, *V. vinifera* and *A. thaliana* (Figure 5). Nearly all of the K_a_/K_s_ ratios were <1.0, suggesting that most of the PCGs were under stabilizing selection during evolution. Combining the information in Figure 5 and Table 1, the K_a_/K_s_ ratios of all Complex I–V genes were <1, indicating that these genes were highly conserved in the evolutionary process of higher plants. The large number of mitochondrial genes under stabilizing selection (K_a_/K_s_ < 1) may play important roles in stabilizing the normal functioning of mitochondria [53,54].

As shown in Figure 5, the K_a_/K_s_ ratios of *ccmB* were >1 between *P. vulgaris* and all of the three selected species, indicating that *ccmB* may have suffered from positive selection since divergence from their last common ancestor. Particularly, the K_a_/K_s_ ratio of *ccmB* between *P. vulgaris* and *V. vinifera* was significantly >1 (4.01), suggesting that some advantage occurred during evolution. Additionally, the K_a_/K_s_ ratios of *ccmF_C_*, *rps1*, *rps10*, and *rps14* were also >1, indicating that these genes were under positive selection after divergence of the last common ancestor. Since *CcmB* and *ccmF_C_* genes encode for some important components of the *c*-type cytochrome maturation pathway in mitochondria, we speculate that the adaptive evolution of *P. vulgaris* is closely related to the roles of *c*-type cytochromes in respiratory and photosynthetic electron transport [55,56,57]. Additionally, *rps1*, *rps10*, and *rps14* genes encode small mitoribosomal subunit proteins, which have been reported to play crucial roles in various biological processes in eukaryotic organisms, such as embryogenesis, leaf morphogenesis, and the formation of reproductive tissues [58,59,60]. The high K_a_/K_s_ ratios of *rps* genes observed here may be very important for the evolution of *P. vulgaris*. K_a_/K_s_ ratios >1 have also been reported for some other mitochondrial genes, including *atp8*, *ccmF_N_*, *matR*, and *mttB* [26,33,61,62], indicating that mitochondrial genes in different plant species may be subjected to diverse selection pressures during evolution. Most importantly, the K_a_/K_s_ ratio of the orthologous gene-pairs is an average over all sites and, even under positive selection, it can be <1 because some sites might be under positive selection while others are under purifying selection [53,61,63].

### 3.4. Prediction of RNA Editing Sites in PCGs

Many previous studies have documented that RNA editing is one of the necessary steps for gene expression in the mitochondrial and chloroplast genomes of higher plants [64,65,66,67]. RNA editing is a post-transcriptional modification that converts specific cytidines (C) to uridines (U) and uridines to uridines in the transcripts of nearly all mitochondrial PCGs. Based on the web-based PREP-mt program, we predicted a total of 486 RNA editing sites in 31 PCGs and 100% C-to-U RNA editing. Among the 486 RNA editing sites, 34.57% (168 sites) were predicted at the first base position of the codon and 65.43% (318 sites) were found in the second position, while none were found in the third position. The lack of predicted RNA editing sites in the silent position is probably due to the limitation of the PREP-Mt predictive methodology rather than there being no RNA editing in this position. Since most of the RNA editing sites in third codon positions did not change the amino acid encoded by the codon, the tie-breaking rules used by PREP-Mt could not select the edited state [68]. Therefore, RNA editing in the silent editing position needs to be further identified by experimental methods.

**Figure 6:**
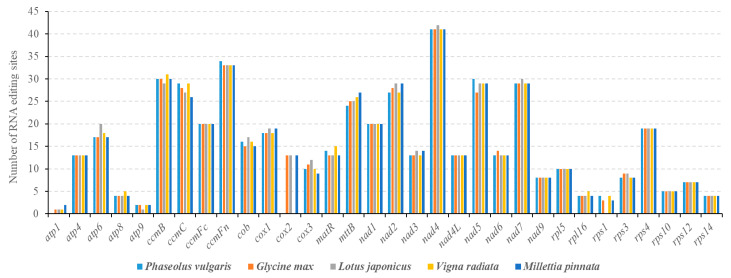
Predicted RNA editing sites of the *P. vulgaris* mitogenome compared with four other leguminous plants. RNA-editing sites are predicted on PREP-Mt sites (http://prep.unl.edu/) with the cut-off value of 0.2.

The occurrence of RNA editing can cause alteration of initiation and termination codons in PCGs, and the frequency of their generation is much higher than that of their removal. As shown in Table 3, *mttB*, *nad1*, *nad4L*, and *rps10* genes use ACG as their initiation codons, which may be altered to the normal AUG by RNA editing modification. Additionally, the *ccmF_C_* gene uses CGA as its termination codon, which may be altered to UGA by RNA editing modification. As shown in Figure 6, the number of RNA editing sites in different genes varies greatly, and the Complex I (NADH dehydrogenase) and Cytochrome *c* biogenesis genes (*ccmB*, *ccmC*, *ccmF_C_*, and *ccmF_N_*) encode the most predicted RNA editing sites. Based on a comparison of the predicted RNA editing sites in five leguminous plants, the *nad4* gene encodes the most RNA editing sites, while *atp1* encodes the fewest (Figure 6).

Previous studies have shown that the frequency and type of RNA editing in each organelle is highly lineage-specific [26,65,69,70]. As shown in Figure 6, the number of predicted RNA editing sites in different papilionoid legume mitogenomes is very conserved, ranging from 486 sites in *P. vulgaris* to 503 sites in *Lotus japonicus*, suggesting that they share extremely conserved PCGs. In angiospermous mitogenomes, nearly all of the RNA editing sites are C to U, and the number of RNA editing sites is concentrated between 400 to 500. For example, 463 and 444 RNA-editing sites were found in the *C. lanatus* and *C. pepo* mitogenomes, of which 394 are shared [8]; 441 and 427 RNA-editing sites were found in *A. thaliana* and *B. napus* mitogenomes, of which 358 are shared [10]. In the gymnosperm *Cycas taitungensis*, 1084 RNA editing sites were found in its mitogenome [71]. The clearly descending number of RNA editing sites is in accordance with gene losses from gymnosperms to angiosperms. In contrast to angiosperms and gymnosperms, both types of C-to-U and U-to-C conversions are found in the mitochondrial transcripts of ferns and hornworts [69,72].

### 3.5. Analysis of Repeat Sequences in the P. vulgaris Mitogenome

The vast majority of the variance in genome size of plant mitogenomes can be explained by differences in the sizes of repeat sequences, which are composed of SSRs, tandem repeats and dispersed repeats. Plant mitogenomes, particularly those of angiosperms, were already well known for its sizeable fractions of repetitive sequences even before any complete mitogenomes were available. SSRs are DNA tracts of tandem-repeated motifs of one to six bases that are useful molecular markers in studying genetic diversity and species identification [21]. In this study, a total of 314 perfect SSRs were identified in the *P. vulgaris* mitogenome, including 139 mono-, 140 di-, 5 tri-, 22 tetra-, 3 penta-, and 5 hexa-nucleotide repeats (Table 4). The mononucleotide repeats of A/T (129 repeats) were found to be more prevalent than other repeat types. The dinucleotides repeats, TA/AT, are the second most numerous (50 repeats), while tri-, tetra-, penta-, and hexa-nucleotide repeats are fewer in number and only observed in intronic or intergenic regions. As shown in Table 5, seven tandem repeats with lengths ranging from 13 bp to 57 bp were also detected in the *P. vulgaris* mitogenome. Among these seven tandem repeats, only one is localized in a coding region (*rrnL*), while the others are all found in intergenic spacers.

**Figure 7:**
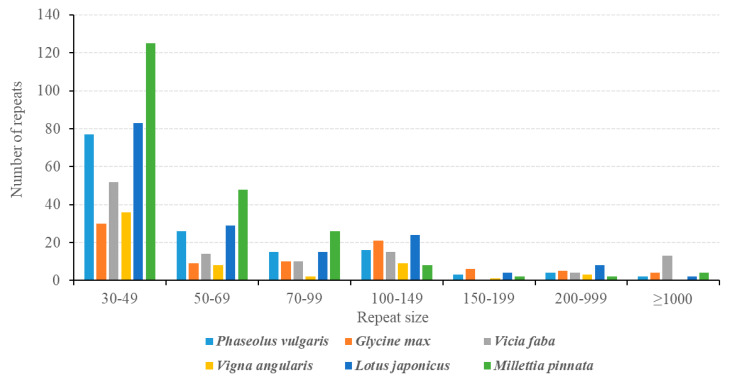
Frequency distribution of dispersed repeat in the *P. vulgaris* mitogenome compared with five other leguminous plants. The number of dispersed repeats in *Phaseolus vulgaris*, *Glycine max Vicia faba*, *Vigna faba*, *Vigna angularis*, *Lotus japonicus*, and *Millettia pinnata* mitogenomes are shown by blue, orange, gray, yellow, blue, and green, respectively.

Besides SSRs and tandem repeats, 143 dispersed repeats with lengths > 30 bp (total length: 35,000 bp; 8.85% of the genome) were also identified in the *P. vulgaris* mitogenome (Figure 7; Table S4). Most of the repeats (77 repeats, 53.85%) are 30 bp to 59 bp long, and 25 repeats are longer than 100 bp, with only two longer than 1 kb (R1: 4866 bp; R2: 3529 bp). Previous studies have documented the importance of large repeats (>1 kb) in genomic structural changes, and pairwise direct and inverted large repeats may produce two small subgenomic conformations or isomeric conformations, respectively. As shown in Figure 1, the largest repeat was assembled as Contig15 and the second largest was assembled as Contig40, both of which were inverted repeats. By aligning the PacBio long reads to both ends of the two large repeats, we constructed the master circle and two isomeric molecules (Figure 1). Repeats are commonly found in plant mitogenomes but are poorly conserved across species, even within the same family. As shown in Figure 7 and Table S4, the total number of repeats ranges from 59 in *V. angularis* to 215 in *M. pinnata*, and the total length of repeats ranges from 9224 bp (2.28% of the whole genome) in *V. angularis* to 411,265 bp (69.94% of the whole genome) in *V. faba*. Mitogenome enlargement in *V. faba* is mainly caused by the expansion of repeated sequences. Thirteen large (>1 kb) repeats covered 398.8 kb or 68% of the whole mitogenome size [17]. However, when all but single copies of the large repeat sequences were excluded, the *V. faba* mitogenome size is 388.6 kb, which is similar to other Papilionoideae mitogenomes [18]. The extremely complex repeat patterns should be responsible for the various genome sizes of the plant mitogenome. However, genome size is by no means only determined by the size of repeats. The mitochondrial genome of *Vitis vinifera* has only 7% repeats despite a genome size of nearly 773 kb [73], while the moderately-sized (404.5 kb) *V. angularis* genome has fewer and smaller repeats than those found in the much smaller genomes of *Brassica napus* (222 kb) and *Silene latifolia* (253 kb; Table S2) [10,74].

### 3.6. Phylogenetic Analyses and Multiple Losses of PCGs during Evolution

**Figure 8:**
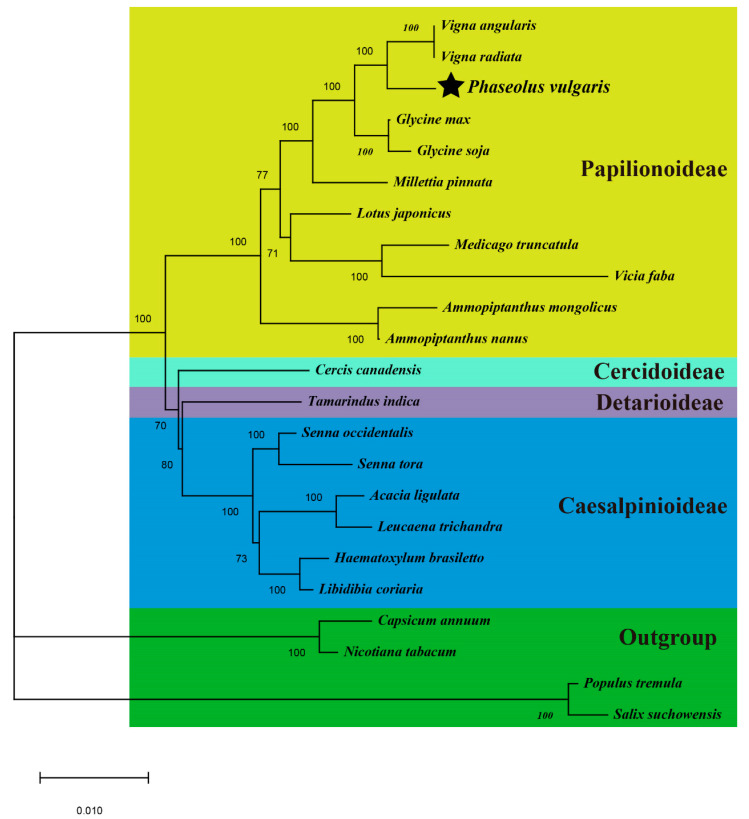
Maximum likelihood phylogenies of *P. vulgaris* within Fabaceae. Relationships were inferred employing 26 conserved PCGs of 23 plant mitogenomes. Numbers on each node are bootstrap support values. NCBI accession numbers are listed in Table S2. Scale indicates number of nucleotide substitutions per site.

With rapid developments in sequencing technology and assembly methods, an increasing number of complete plant mitogenomes has been assembled, providing an important opportunity for phylogenetic analyses using mitogenomes. In this study, to determine the phylogenetic position of *P. vulgaris*, we downloaded 23 plant mitogenomes from the GenBank database (https://www.ncbi.nlm.nih.gov/genome/browse/), including 19 species of Fabales, two species of Solanales, and two species of Malpighiales. A set of 26 conserved single-copy orthologous genes (*atp1*, *atp4*, *atp6*, *atp8*, *atp9*, *ccmB*, *ccmC*, *ccmF_C_*, *ccmF_N_*, *cob*, *cox1*, *cox3*, *matR*, *nad1*, *nad2*, *nad3*, *nad4*, *nad4L*, *nad5*, *nad6*, *nad7*, *nad9*, *rps3*, *rps4*, and *rps12*) present in all of the 23 analyzed mitogenomes was used to construct the phylogenetic tree, and species from the Solanales and Malpighiales were designated as the outgroup. As shown in Figure 8, the bootstrap values of each node are all over 70% supported and 15 nodes are supported 100%. The ML phylogenetic tree strongly supports that *P. vulgaris* is evolutionarily close to the clade formed by two Vigna species. The tree also strongly supports the separation of Fabales from the clade composed of Solanales and Malpighiales (100% bootstrap value), as well as the separation of Papilionoideae from the clade composed of Cercidoideae, Detarioideae, and Caesalpinioideae (100%). The bootstrap value for the separation of Detarioideae and Caesalpinioideae is 80%, and the value for the separation of Cercidoideae from the clade composed of Detarioideae and Caesalpinioideae is 70%.

**Figure 9:**
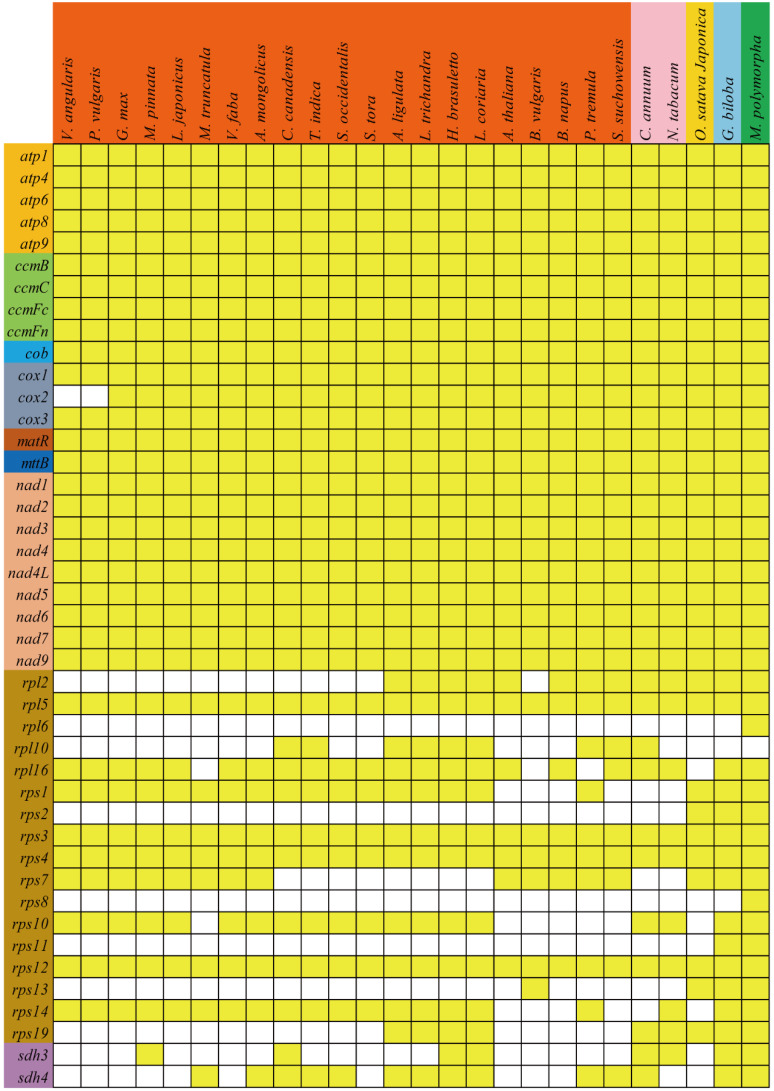
Distribution of PCGs in plant mitogenomes. White boxes indicate that the gene is not present in the mitogenome. The colors of genes indicate their corresponding categories. The colors of species represent the classes of rosids (orange), asterids (pink), monocotyledons (gold), gymnosperms (light blue), and liverworts (green).

As described by Richardson et al. [75], the mitochondrial genomes of higher plants vary significantly in genome size, gene content and order. Losses of PCGs occurred frequently during the evolution of higher plants. The phylogenetic tree provides a backdrop for the further analysis of gene loss during evolution, and the gene contents of all observed species are summarized in Figure 9. Most of the PCGs were conserved in different plant mitogenomes, especially for the genes in the groups of Complex I, Complex III, Complex V, cytochrome *c* biogenesis, maturases, and transport membrane protein [13]. The conservation of these genes suggests that they play crucial roles in the function of mitochondria. However, the ribosomal proteins and succinate dehydrogenase genes were highly variable. As shown in Figure 9, the *cox2* gene was only lost in the subfamily Phaseolinae (*V. angularis*, *V. radiata*, and *P. vulgaris*) but retained in other leguminous plants, suggesting that this gene was lost after separation from the subfamily Glycininae. The *rpl2* gene was lost in most leguminous plants but regained in *A. ligulate*, *L. trichandra*, *H. brasuletto*, and *L. coriaria*, suggesting that this gene was lost before the emergence of Fabales but could be regained in some leguminous plants. Similar phenomena were found in many ribosomal proteins (*rpl10*, *rpl16*, *rps7*, *rps10*, and *rps19*). Additionally, *rpl6* and *rps8* genes were lost from liverworts (*M. polymorpha*) during evolution [76], the *rps11* gene was lost from gymnosperms (*G. biloba*) and liverworts during the divergence of the angiosperms and gymnosperms [77], and the *rpl10* gene was lost in monocots and gymnosperms but regained in dicots [33,78]. The enhanced loss of ribosomal proteins in plant mitogenomes indicates that these genes were encoded partly by mitochondrial native genes and partly by nuclear genes, due to the gene transfer between mitochondria and nucleus [79,80,81].

## 4. Conclusions

In this study, we first assembled and characterized the complete mitogenome of *P. vulgaris*. By aligning the PacBio sequencing reads to the draft mitogenome, one master circle and two isomeric molecules were assembled based on two large repeats. Selective-pressure analysis of PCGs indicates that *ccmB*, *ccmF_C_*, *rps1*, *rps10*, and *rps14* genes with K_a_/K_s_ ratios > 1 might play important roles during evolution, whereas all Complex I–V genes with K_a_/K_s_ ratios < 1 were highly conserved in the evolutionary process of higher plants. The C-to-U conversions may generate initiation, termination, or internal codons with completely unpredictable functions. The prediction of RNA editing sites in *P. vulgaris* mt PCGs will provide important clues for the investigation of gene functions with novel codons. The comparison of genomic features in all sequenced leguminous plants should contribute to a comprehensive understanding of the evolutionary process of legumes. The sequencing of the *P. vulgaris* mitogenome not only provides an important opportunity to conduct further genomic breeding studies in the common bean, it also provides valuable information for future evolutionary and molecular studies of leguminous plants.

## Figures and Tables

**Table 1:** Genomic features of *P. vulgaris* mitogenome.

Feature	A %	C %	G %	T %	GC %	Size (bp)	Proportion in Genome (%)
Whole genome	27.37	22.40	22.71	27.52	45.11	395,516	100
Protein-coding genes ^a^	26.34	21.29	21.62	30.75	42.91	28,725	7.26
*cis*-spliced introns ^a^	24.49	25.56	24.79	25.16	50.34	32,584	8.24
tRNA genes ^a^	23.07	24.34	27.49	25.09	51.84	1335	0.34
rRNA genes ^a^	26.14	22.62	29.04	22.20	51.66	5252	1.33
Non-coding regions	27.78	22.18	22.48	27.56	44.65	327,620	82.83

^a^ Protein-coding genes, *cis*-spliced introns, tRNAs, and rRNAs belong to coding regions.

**Table 2:** Gene content of *P. vulgaris* mitogenome.

Group of Genes	Gene Name
Complex I (NADH dehydrogenase)	*nad1* *, *nad2* *, *nad3*, *nad4* *, *nad4L*, *nad5* *, *nad6*, *nad7* *, *nad9*
Complex II (succinate dehydrogenase)	-
Complex III (ubiquinol cytochrome c reductase)	*cob*
Complex IV (cytochrome c oxidase)	*cox1*, *cox3*
Complex V (ATP synthase)	*atp1*, *atp4*, *atp6*, *atp8*, *atp9*
Cytochrome *c* biogenesis	*ccmB*, *ccmC*, *ccmF_C_* *, *ccmF_N_*
Ribosomal proteins (SSU)	*rps1*, *rps3* *, *rps4*, *rps10* *, *rps12*, *rps14*
Ribosomal proteins (LSU)	*rpl5*, *rpl16*
Maturases	*matR*
Transport membrane protein	*mttB*
Ribosomal RNAs	*rrn5*, *rrnS*, *rrnL*
Transfer RNAs	*trnC-GCA* (2 copies), *trnD-GUC*, *trnE-UUC*, *trnF-GAA*, *trnG-GCC*, *trnfM-CAU* (3 copies), *trnH-GUG*, *trnI-CAU*, *trnK-UUU*, *trnM-CAU*, *trnN-GUU*, *trnP-UGG*, *trnQ-UUG*, *trnW-CCA*, *trnY-GUA*

* The asterisks besides genes denotes intron-containing genes.

**Table 3:** Gene profile and organization of PCGs in *P. vulgaris* mitogenome.

Gene Name	Length	Start Codon	Stop Codon	Direction
*atp1*	1527	ATG	TGA	F
*atp4*	588	ATG	TAA	F
*atp6*	726	ATG	TAG	F
*atp8*	483	ATG	TAA	R
*atp9*	225	ATG	TAA	R
*ccmB*	621	ATG	TGA	F
*ccmC*	741	ATG	TGA	R
*ccmF_C_*	1329	ATG	CGA	F
*ccmF_N_*	1740	ATG	TGA	R
*cob*	1176	ATG	TAG	F
*cox1*	1584	ATG	TAA	F
*cox3*	798	ATG	TGA	F
*matR*	2010	ATG	TGA	F
*mttB*	723	ACG	TGA	F
*nad1*	978	ACG	TAA	F
*nad2*	1467	ATG	TAA	R
*nad3*	357	ATG	TAA	R
*nad4*	1488	ATG	TGA	R
*nad4L*	303	ACG	TAA	F
*nad5*	2019	ATG	TAA	F/R
*nad6*	618	ATG	TAA	F
*nad7*	1185	ATG	TAG	R
*nad9*	573	ATG	TAA	F
*rpl5*	558	ATG	TAA	F
*rpl16*	516	ATG	TAA	F
*rps1*	618	ATG	TAA	R
*rps3*	1689	ATG	TAG	F
*rps4*	1041	ATG	TAA	F
*rps10*	363	ACG	TGA	F
*rps12*	378	ATG	TGA	R
*rps14*	303	ATG	TAG	F

**Table 4:** Frequency of identified SSR motifs in *P. vulgaris* mitogenome.

Motif Type	Number of Repeats											Total	Proportion (%)
	3	4	5	6	7	8	9	10	11	12	13		
Monomer	-	-	-	-	-	91	32	10	4	1	1	139	44.27
Dimer	-	120	16	4	0	0	0	0	0	0	0	140	44.59
Trimer	-	5	0	0	0	0	0	0	0	0	0	5	1.59
Tetramer	21	1	0	0	0	0	0	0	0	0	0	22	7.01
Pentamer	3	0	0	0	0	0	0	0	0	0	0	3	0.96
Hexamer	5	0	0	0	0	0	0	0	0	0	0	5	1.59
Total	29	126	16	4	0	91	32	10	4	1	1	314	100

**Table 5:** Distribution of tandem repeats in *P. vulgaris* mitogenome.

No	Size (bp)	Start	End	Repeat (bp) × Copy Number	Location
1	57	75,208	75,322	(TTGGATCAAAACGATGTTCAACAACCTTTGCCGCGTCTGTTTCTTGGAGGAAAATAG) × 2	IGS (*trnD*, *atp6*)
2	27	92,189	92,243	(AGAGCAGGTCGGTCTAGGTAGTTGAAA) × 2	IGS (*trnC*, *trnN*)
3	38	108,846	108,922	(AAAAATATACATAACATATCCCAAACTCTATAGAGATA) × 2	IGS (*rrn5*, *ccmF_N_*)
4	13	231,727	231,753	(TCTTAAGTAAAGT) × 2	IGS (*nad2*-exon1, *trnH*)
5	18	252,332	252,368	(CATAGTCGCGAGCTGTTT) × 2	rrnL
6	15	315,763	315,793	(GTATAGTATAGTAGG) × 2	IGS (*nad1*-exon1, *nad6*)
7	33	356,560	356,626	(CCTTGCCCCCTGCAGAGCCTCAAGCCCCTGAGC) × 2	IGS (*atp1*, *nad4L*)
IGS: intergenic pacers.					

## Supplementary Materials

The following are available online at https://www.mdpi.com/1422-0067/21/11/3778/s1, Figure S1: The flowchart of *P. vulgaris* mitogenome assembly and annotation, Table S1: PacBio reads of spanning repetitive contigs, Table S2: The abbreviations and genome sizes of studied mitogenomes, Table S3: The relative synonymous codon usage (RSCU) of mitogenome genome across seven higher plants, Table S4: Comparison of dispersed repeats within six mitogenomes of Papilionaceae subfamily.

## References

[1] Greiner S., Bock R. (2013). Tuning a menage a trois: Co-evolution and co-adaptation of nuclear and organellar genomes in plants. Bioessays.

[2] Timmis J.N., Ayliffe M.A., Huang C.Y., Martin W. (2004). Endosymbiotic gene transfer: Organelle genomes forge eukaryotic chromosomes. Nat. Rev. Genet..

[3] Hsu C.L., Mullin B.C. (1989). Physical characterization of mitochondrial DNA from cotton. Plant Mol. Biol..

[4] Kubo T., Mikami T. (2007). Organization and variation of angiosperm mitochondrial genome. Physiol. Plant..

[5] Palmer J.D., Herbon L.A. (1988). Plant mitochondrial DNA evolves rapidly in structure, but slowly in sequence. J. Mol. Evol..

[6] Gray M.W., Burger G., Lang B.F. (1999). Mitochondrial Evolution. Science.

[7] Lang B.F., Gray M.W., Burger G. (1999). Mitochondrial Genome Evolution and the Origin of Eukaryotes. Annu. Rev. Genet..

[8] Alverson A.J., Wei X.X., Rice D.W., Stern D.B., Barry K., Palmer J.D. (2010). Insights into the Evolution of Mitochondrial Genome Size from Complete Sequences of *Citrullus lanatus* and *Cucurbita pepo* (Cucurbitaceae). Mol. Biol. Evol..

[9] Alverson A.J., Zhuo S., Rice D.W., Sloan D.B., Palmer J.D. (2011). The mitochondrial genome of the legume *Vigna radiata* and the analysis of recombination across short mitochondrial repeats. PLoS ONE.

[10] Handa H. (2003). The complete nucleotide sequence and RNA editing content of the mitochondrial genome of rapeseed (*Brassica napus* L.): Comparative analysis of the mitochondrial genomes of rapeseed and *Arabidopsis thaliana*. Nucleic Acids Res..

[11] Mulligan R.M., Chang K.L., Chou C.C. (2007). Computational analysis of RNA editing sites in plant mitochondrial genomes reveals similar information content and a sporadic distribution of editing sites. Mol. Biol. Evol..

[12] Andre C., Levy A., Walbot V. (1992). Small repeated sequences and the structure of plant mitochondrial genomes. Trends Genet..

[13] Chang S., Wang Y., Lu J., Gai J., Li J., Chu P., Guan R., Zhao T. (2013). Correction: The Mitochondrial Genome of Soybean Reveals Complex Genome Structures and Gene Evolution at Intercellular and Phylogenetic Levels. PLoS ONE.

[14] Skippington E., Barkman T.J., Rice D.W., Palmer J.D. (2015). Miniaturized mitogenome of the parasitic plant *Viscum scurruloideum* is extremely divergent and dynamic and has lost all nad genes. Proc. Natl. Acad. Sci. USA.

[15] Sloan D.B., Alverson A.J., Chuckalovcak J.P., Wu M., McCauley D.E., Palmer J.D., Taylor D.R. (2012). Rapid evolution of enormous, multichromosomal genomes in flowering plant mitochondria with exceptionally high mutation rates. PLoS Biol..

[16] Bi C., Wang X., Xu Y., Wei S., Shi Y., Dai X., Yin T., Ye N. (2016). The complete mitochondrial genome of *Medicago truncatula*. Mitochondrial DNA Part B.

[17] Negruk V. (2013). Mitochondrial genome sequence of the legume *Vicia faba*. Front. Plant Sci..

[18] Choi I.-S., Schwarz E.N., Ruhlman T.A., Khiyami M.A., Sabir J.S., Hajarah N.H., Sabir M.J., Rabah S.O., Jansen R.K. (2019). Fluctuations in Fabaceae mitochondrial genome size and content are both ancient and recent. BMC Plant Biol..

[19] Wynn E.L., Christensen A.C. (2019). Repeats of Unusual Size in Plant Mitochondrial Genomes: Identification, Incidence and Evolution. G3 Genes Genomes Genet..

[20] Bergthorsson U., Adams K.L., Thomason B., Palmer J.D. (2003). Widespread horizontal transfer of mitochondrial genes in flowering plants. Nature.

[21] Ma Q., Li S., Bi C., Hao Z., Sun C., Ye N. (2017). Complete chloroplast genome sequence of a major economic species, *Ziziphus jujuba* (Rhamnaceae). Curr. Genet..

[22] Sperisen C., Büchler U., Gugerli F., Mátyás G., Geburek T., Vendramin G. (2001). Tandem repeats in plant mitochondrial genomes: Application to the analysis of population differentiation in the conifer Norway spruce. Mol. Ecol..

[23] Alverson A.J., Rice D.W., Dickinson S., Barry K., Palmer J.D. (2011). Origins and Recombination of the Bacterial-Sized Multichromosomal Mitochondrial Genome of Cucumber. Plant Cell.

[24] Backert S., Nielsen B.L., Börner T. (1997). The mystery of the rings: Structure and replication of mitochondrial genomes from higher plants. Trends Plant Sci..

[25] Ogihara Y., Yamazaki Y., Murai K., Kanno A., Terachi T., Shiina T., Miyashita N., Nasuda S., Nakamura C., Mori N. (2005). Structural dynamics of cereal mitochondrial genomes as revealed by complete nucleotide sequencing of the wheat mitochondrial genome. Nucleic Acids Res..

[26] Bi C., Paterson A.H., Wang X., Xu Y., Wu D., Qu Y., Jiang A., Ye Q., Ye N. (2016). Analysis of the complete mitochondrial genome sequence of the diploid cotton *Gossypium raimondii* by comparative genomics approaches. BioMed. Res. Int..

[27] Christenhusz M.J., Byng J.W. (2016). The number of known plants species in the world and its annual increase. Phytotaxa.

[28] Azani N., Babineau M., Bailey C.D., Banks H., Barbosa A.R., Pinto R.B., Boatwright J.S., Borges L.M., Brown G.K., Bruneau A. (2017). A new subfamily classification of the Leguminosae based on a taxonomically comprehensive phylogeny: The Legume Phylogeny Working Group (LPWG). Taxon.

[29] Schmutz J., McClean P.E., Mamidi S., Wu G.A., Cannon S.B., Grimwood J., Jenkins J., Shu S., Song Q., Chavarro C. (2014). A reference genome for common bean and genome-wide analysis of dual domestications. Nat. Genet..

[30] Fonsêca A., Ferreira J., dos Santos T.R.B., Mosiolek M., Bellucci E., Kami J., Gepts P., Geffroy V., Schweizer D., dos Santos K.G. (2010). Cytogenetic map of common bean (*Phaseolus vulgaris* L.). Chrom. Res..

[31] Zhang T., Fang Y., Wang X., Deng X., Zhang X., Hu S., Yu J. (2012). The Complete Chloroplast and Mitochondrial Genome Sequences of *Boea hygrometrica*: Insights into the Evolution of Plant Organellar Genomes. PLoS ONE.

[32] Iorizzo M., Senalik D., Szklarczyk M., Grzebelus D., Spooner D., Simon P. (2012). *De novo* assembly of the carrot mitochondrial genome using next generation sequencing of whole genomic DNA provides first evidence of DNA transfer into an angiosperm plastid genome. BMC Plant Biol..

[33] Ye N., Wang X., Li J., Bi C., Xu Y., Wu D., Ye Q. (2017). Assembly and comparative analysis of complete mitochondrial genome sequence of an economic plant *Salix suchowensis*. PeerJ.

[34] Nederbragt A.J. (2014). On the middle ground between open source and commercial software-the case of the Newbler program. Genome Biol..

[35] Camacho C., Coulouris G., Avagyan V., Ma N., Papadopoulos J., Bealer K., Madden T.L. (2009). BLAST+: Architecture and applications. BMC Bioinform..

[36] Zhang T., Zhang X., Hu S., Yu J. (2011). An efficient procedure for plant organellar genome assembly, based on whole genome data from the 454 GS FLX sequencing platform. Plant Methods.

[37] Dong S., Zhao C., Chen F., Liu Y., Zhang S., Wu H., Zhang L., Liu Y. (2018). The complete mitochondrial genome of the early flowering plant *Nymphaea colorata* is highly repetitive with low recombination. BMC Genom..

[38] Li H., Durbin R. (2009). Fast and accurate short read alignment with Burrows–Wheeler transform. Bioinformatics.

[39] Li H., Handsaker B., Wysoker A., Fennell T., Ruan J., Homer N., Marth G., Abecasis G., Durbin R. (2009). The Sequence Alignment/Map format and SAMtools. Bioinformatics.

[40] Schattner P., Brooks A.N., Lowe T.M. (2005). The tRNAscan-SE, snoscan and snoGPS web servers for the detection of tRNAs and snoRNAs. Nucleic Acids Res..

[41] Kumar S., Stecher G., Li M., Knyaz C., Tamura K. (2018). MEGA X: Molecular Evolutionary Genetics Analysis across Computing Platforms. Mol. Biol. Evol..

[42] Greiner S., Lehwark P., Bock R. (2019). OrganellarGenomeDRAW (OGDRAW) version 1.3. 1: Expanded toolkit for the graphical visualization of organellar genomes. Nucleic Acids Res..

[43] Zhang Z., Xiao J., Wu J., Zhang H., Liu G., Wang X., Dai L. (2012). ParaAT: A parallel tool for constructing multiple protein-coding DNA alignments. Biochem. Biophys. Res. Commun..

[44] Yang Z., Nielsen R. (2000). Estimating Synonymous and Nonsynonymous Substitution Rates Under Realistic Evolutionary Models. Mol. Biol. Evol..

[45] Wang D., Zhang Y., Zhang Z., Zhu J., Yu J. (2010). KaKs_Calculator 2.0: A Toolkit Incorporating Gamma-Series Methods and Sliding Window Strategies. Genom. Proteom. Bioinform..

[46] Mower J.P. (2009). The PREP suite: Predictive RNA editors for plant mitochondrial genes, chloroplast genes and user-defined alignments. Nucleic Acids Res..

[47] Beier S., Thiel T., Münch T., Scholz U., Mascher M. (2017). MISA-web: A web server for microsatellite prediction. Bioinformatics.

[48] Benson G. (1999). Tandem repeats finder: A program to analyze DNA sequences. Nucleic Acids Res..

[49] Liu G., Cao D., Li S., Su A., Geng J., Grover C.E., Hu S., Hua J. (2013). The Complete Mitochondrial Genome of *Gossypium hirsutum* and Evolutionary Analysis of Higher Plant Mitochondrial Genomes. PLoS ONE.

[50] Edgar R.C. (2004). MUSCLE: Multiple sequence alignment with high accuracy and high throughput. Nucleic Acids Res..

[51] Shen Y., Du H., Liu Y., Ni L., Wang Z., Liang C., Tian Z. (2019). Update soybean Zhonghuang 13 genome to a golden reference. Sci. China Life Sci..

[52] Li W.-H., Wu C.-I., Luo C.-C. (1985). A new method for estimating synonymous and nonsynonymous rates of nucleotide substitution considering the relative likelihood of nucleotide and codon changes. Mol. Biol. Evol..

[53] Betrán E., Bai Y., Motiwale M. (2006). Fast Protein Evolution and Germ Line Expression of a Drosophila Parental Gene and Its Young Retroposed Paralog. Mol. Biol. Evol..

[54] Arbiza L., Dopazo J., Dopazo H. (2006). Positive Selection, Relaxation, and Acceleration in the Evolution of the Human and Chimp Genome. PLoS Comp. Biol..

[55] Meyer E.H., Giegé P., Gelhaye E., Rayapuram N., Ahuja U., Thöny-Meyer L., Grienenberger J.-M., Bonnard G. (2005). AtCCMH, an essential component of the *c*-type cytochrome maturation pathway in *Arabidopsis* mitochondria, interacts with apocytochrome *c*. Proc. Natl. Acad. Sci. USA.

[56] Faivre-Nitschke S.E., Nazoa P., Gualberto J.M., Grienenberger J.M., Bonnard G. (2001). Wheat mitochondria *ccmB* encodes the membrane domain of a putative ABC transporter involved in cytochrome *c* biogenesis. Biochim. Biophys. Acta.

[57] Sanders C., Turkarslan S., Lee D.-W., Daldal F. (2010). Cytochrome *c* biogenesis: The Ccm system. Trends Microbiol..

[58] Robles P., Quesada V. (2017). Emerging Roles of Mitochondrial Ribosomal Proteins in Plant Development. Int. J. Mol. Sci..

[59] Mauro V.P., Edelman G.M. (2007). The Ribosome Filter Redux. Cell Cycle.

[60] Schippers J.H.M., Mueller-Roeber B. (2010). Ribosomal composition and control of leaf development. Plant Sci..

[61] Cui P., Liu H., Lin Q., Ding F., Zhuo G., Hu S., Liu D., Yang W., Zhan K., Zhang A. (2009). A complete mitochondrial genome of wheat (*Triticum aestivum* cv. Chinese Yumai), and fast evolving mitochondrial genes in higher plants. J. Genet..

[62] Feng L., Li N., Yang W., Li Y., Wang C.-M., Tong S.-W., He J.-X. (2019). Analyses of mitochondrial genomes of the genus *Ammopiptanthus* provide new insights into the evolution of legume plants. Plant Syst. Evol..

[63] Wernegreen J.J., Riley M.A. (1999). Comparison of the evolutionary dynamics of symbiotic and housekeeping loci: A case for the genetic coherence of rhizobial lineages. Mol. Biol. Evol..

[64] Bock R., Khan M.S. (2004). Taming plastids for a green future. Trends Biotechnol..

[65] Chen H., Deng L., Jiang Y., Lu P., Yu J. (2011). RNA Editing Sites Exist in Protein-coding Genes in the Chloroplast Genome of *Cycas taitungensis*. J. Integr. Plant Biol..

[66] Raman G., Park S. (2015). Analysis of the Complete Chloroplast Genome of a Medicinal Plant, *Dianthus superbus* var. longicalyncinus, from a Comparative Genomics Perspective. PLoS ONE.

[67] Wakasugi T., Hirose T., Horihata M., Tsudzuki T., Kössel H., Sugiura M. (1996). Creation of a novel protein-coding region at the RNA level in black pine chloroplasts: The pattern of RNA editing in the gymnosperm chloroplast is different from that in angiosperms. Proc. Natl. Acad. Sci. USA.

[68] Mower J.P. (2005). PREP-Mt: Predictive RNA editor for plant mitochondrial genes. BMC Bioinform..

[69] Malek O., Lättig K., Hiesel R., Brennicke A., Knoop V. (1996). RNA editing in bryophytes and a molecular phylogeny of land plants. EMBO J..

[70] Steinhauser S., Beckert S., Capesius I., Malek O., Knoop V. (1999). Plant Mitochondrial RNA Editing. J. Mol. Evol..

[71] Shu-Miaw C., Arthur C.C.S., Wang D., Yu-Wei W., Shu-Mei L., The-Yuan C. (2008). The Mitochondrial Genome of the Gymnosperm *Cycas taitungensis* Contains a Novel Family of Short Interspersed Elements, Bpu Sequences, and Abundant RNA Editing Sites. Mol. Biol. Evol..

[72] Hiesel R., Combettes B., Brennicke A. (1994). Evidence for RNA editing in mitochondria of all major groups of land plants except the Bryophyta. Proc. Natl. Acad. Sci. USA.

[73] Goremykin V.V., Salamini F., Velasco R., Viola R. (2009). Mitochondrial DNA of *Vitis vinifera* and the Issue of Rampant Horizontal Gene Transfer. Mol. Biol. Evol..

[74] Sloan D.B., Alverson A.J., Štorchová H., Palmer J.D., Taylor D.R. (2010). Extensive loss of translational genes in the structurally dynamic mitochondrial genome of the angiosperm *Silene latifolia*. BMC Evol. Biol..

[75] Richardson A.O., Rice D.W., Young G.J., Alverson A.J., Palmer J.D. (2013). The “fossilized” mitochondrial genome of *Liriodendron tulipifera*: Ancestral gene content and order, ancestral editing sites, and extraordinarily low mutation rate. BMC Biol..

[76] Bowman J.L., Kohchi T., Yamato K.T., Jenkins J., Shu S. (2017). Insights into Land Plant Evolution Garnered from the *Marchantia polymorpha* Genome. Cell.

[77] Guo W., Felix G., Fan W., Young G.J., Volker K., Palmer J.D., Mower J.P. (2016). *Ginkgo* and *Welwitschia* Mitogenomes Reveal Extreme Contrasts in Gymnosperm Mitochondrial Evolution. Mol. Biol. Evol..

[78] Notsu Y., Masood S., Nishikawa T., Kubo N., Akiduki G., Nakazono M., Hirai A., Kadowaki K. (2002). The complete sequence of the rice (*Oryza sativa* L.) mitochondrial genome: Frequent DNA sequence acquisition and loss during the evolution of flowering plants. Mol. Genet. Genom..

[79] Clifton S.W., Minx P., Fauron C.M.-R., Gibson M., Allen J.O., Sun H., Thompson M., Barbazuk W.B., Kanuganti S., Tayloe C. (2004). Sequence and Comparative Analysis of the Maize NB Mitochondrial Genome. Plant Physiol..

[80] Unseld M., Marienfeld J.R., Brandt P., Brennicke A. (1997). The mitochondrial genome of *Arabidopsis thaliana* contains 57 genes in 366,924 nucleotides. Nat. Genet..

[81] Sugiyama Y., Watase Y., Nagase M., Makita N., Yagura S., Hirai A., Sugiura M. (2005). The complete nucleotide sequence and multipartite organization of the tobacco mitochondrial genome: Comparative analysis of mitochondrial genomes in higher plants. Mol. Genet. Genom..

