# Supplementary material for: Characterization and Analysis of the Mitochondrial Genome of Common Bean (Phaseolus vulgaris) by Comparative Genomic Approaches
Source: Int J Mol Sci. 2020 May 27;21(11):3778. doi: 10.3390/ijms21113778 (PMC7312688; doi:10.3390/ijms21113778)
Supplement: Supplementary file 1 [file ijms-21-03778-s001.pdf]

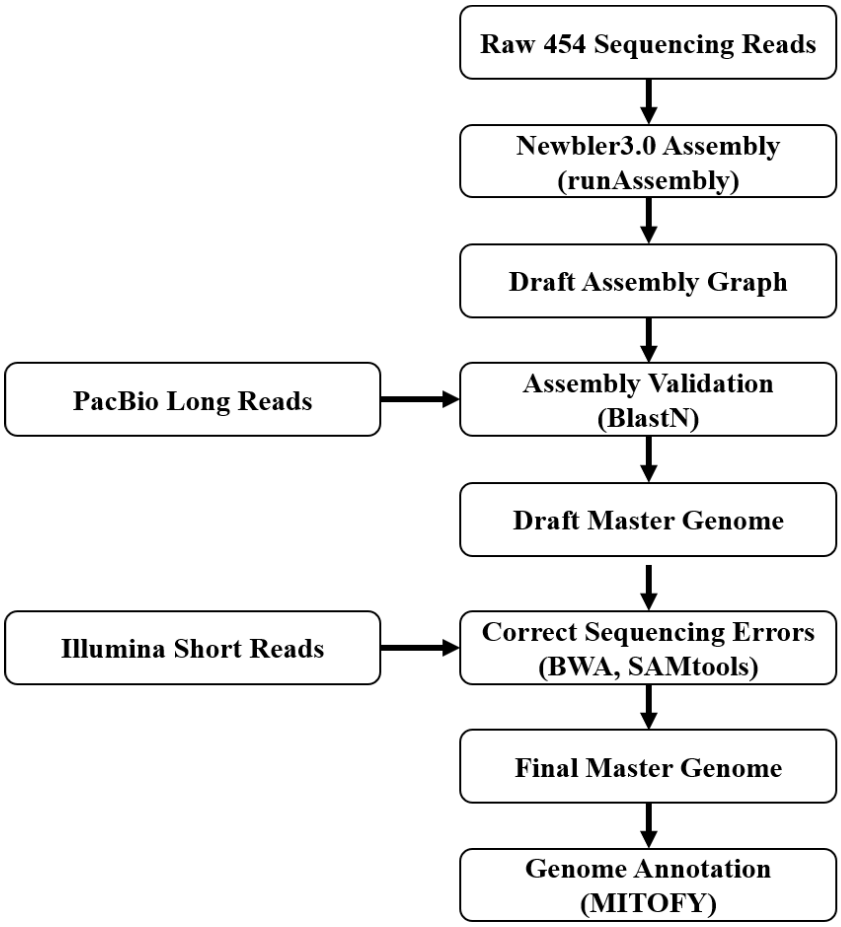

Figure S1. The flowchart of *P. vulgaris* mitogenome assembly and annotation. Raw 454 sequencing reads were used to construct the draft assembly graph, PacBio long reads were used to validate the master genome, and Illumina short reads were used to correct sequencing errors.

.

Tables.

Table 1. Genomic features of *P. vulgaris* mitogenome.

Feature.	A %.	C %.	G %.	T %.	GC %.	Size (bp).	Proportion in genome (%)..
Whole genome.	27.37 .	22.40 .	22.71 .	27.52 .	45.11 .	395,516 .	100 ..
Protein-coding genes[a.]	26.34 .	21.29 .	21.62 .	30.75 .	42.91 .	28,725 .	7.26 ..
cis-spliced introns[a.]	24.49 .	25.56 .	24.79 .	25.16 .	50.34 .	32,584 .	8.24 ..
tRNA genes[a.]	23.07 .	24.34 .	27.49 .	25.09 .	51.84 .	1,335 .	0.34 ..
rRNA genes[a.]	26.14 .	22.62 .	29.04 .	22.20 .	51.66 .	5,252 .	1.33 ..
Non-coding regions.	27.78 .	22.18 .	22.48 .	27.56 .	44.65 .	327,620 .	82.83 ..

[a]Protein-coding genes, cis-spliced introns, tRNAs, and rRNAs belong to coding regions.

..

Table 2. Gene content of *P. vulgaris* mitogenome.

Group of genes.	Gene name..
Complex I (NADH dehydrogenase).	*nad1**, *nad2**, *nad3*, *nad4**, *nad4L*, *nad5**, *nad6*, *nad7**, *nad9*..
Complex II (succinate dehydrogenase).	-..
Complex III (ubiquinol cytochrome c reductase).	*cob*..
Complex IV (cytochrome c oxidase).	*cox1*, *cox3*..
Complex V (ATP synthase).	*atp1*, *atp4*, *atp6*, *atp8*, *atp9*..
Cytochrome *c* biogenesis.	*ccmB*, *ccmC*, *ccmF$_C$**, *ccmF$_N$*..
Ribosomal proteins (SSU).	*rps1*, *rps3**, *rps4*, *rps10**, *rps12*, *rps14*..
Ribosomal proteins (LSU).	*rpl5*, *rpl16*..
Maturases.	*matR*..
Transport membrane protein.	*mttB*..
Ribosomal RNAs.	*rrn5*, *rrnS*, *rrnL*..
Transfer RNAs.	*trnC-GCA* (2 copies), *trnD-GUC*, *trnE-UUC*, *trnF-GAA*, *trnG-GCC*, *trnfM-CAU* (3 copies), *trnH-GUG*, *trnI-CAU*, *trnK-UUU*, *trnM-CAU*, *trnN-GUU*, *trnP-UGG*, *trnQ-UUG*, *trnW-CCA*, *trnY-GUA*..

[*] The asterisks besides genes denotes intron-containing genes.

Table 3. Gene profile and organization of PCGs in *P. vulgaris* mitogenome.

Gene name.	Length.	Start codon.	Stop codon.	Direction..
atp1.	1527.	ATG.	TGA.	F..
atp4.	588.	ATG.	TAA.	F..
atp6.	726.	ATG.	TAG.	F..
atp8.	483.	ATG.	TAA.	R..
atp9.	225.	ATG.	TAA.	R..
ccmB.	621.	ATG.	TGA.	F..
ccmC.	741.	ATG.	TGA.	R..
$ccmF_{C.}$	1329.	ATG.	CGA.	F..
$ccmF_{N.}$	1740.	ATG.	TGA.	R..
cob.	1176.	ATG.	TAG.	F..
cox1.	1584.	ATG.	TAA.	F..
cox3.	798.	ATG.	TGA.	F..
matR.	2010.	ATG.	TGA.	F..
mttB.	723.	ACG.	TGA.	F..
nad1.	978.	ACG.	TAA.	F..
nad2.	1467.	ATG.	TAA.	R..
nad3.	357.	ATG.	TAA.	R..
nad4.	1488.	ATG.	TGA.	R..
nad4L.	303.	ACG.	TAA.	F..
nad5.	2019.	ATG.	TAA.	F/R..
nad6.	618.	ATG.	TAA.	F..
nad7.	1185.	ATG.	TAG.	R..
nad9.	573.	ATG.	TAA.	F..
rpl5.	558.	ATG.	TAA.	F..
rpl16.	516.	ATG.	TAA.	F..
rps1.	618.	ATG.	TAA.	R..
rps3.	1689.	ATG.	TAG.	F..
rps4.	1041.	ATG.	TAA.	F..
rps10.	363.	ACG.	TGA.	F..
rps12.	378.	ATG.	TGA.	R..
rps14.	303.	ATG.	TAG.	F..

.

Table 4. Frequency of identified SSR motifs in *P. vulgaris* mitogenome.

Motif type.	Number of Repeats.											Total.	Proportion (%)..
	3	4	5	6	7	8	9	10	11	12	13		
Monomer	-	-	-	-	-	91	32	10	4	1	1	139	44.27
Dimer	-	120	16	4	0	0	0	0	0	0	0	140	44.59
Trimer	-	5	0	0	0	0	0	0	0	0	0	5	1.59
Tetramer	21	1	0	0	0	0	0	0	0	0	0	22	7.01
Pentamer	3	0	0	0	0	0	0	0	0	0	0	3	0.96
Hexamer	5	0	0	0	0	0	0	0	0	0	0	5	1.59
Total	29	126	16	4	0	91	32	10	4	1	1	314	100

Table 5. Distribution of tandem repeats in *P. vulgaris* mitogenome

No.	Size (bp)	Start	End	Repeat (bp) × copy number	Location
1	57	75,208	75,322	(TTGGATCAAAACGATGTTCAACAACCTTTGCC GCGTCTGTTTCTTGGAGGAAAATAG)×2	IGS (*trnD*, *atp6*)
2	27	92,189	92,243	(AGAGCAGGTCGGTCTAGGTAGTTGAAA)×2	IGS (*trnC*, *trnN*)
3	38	108,846	108,922	(AAAAATATACATAACATATCCCAAACTCTAT AGAGATA)×2	IGS (*rrn5*, *ccmF*N)
4	13	231,727	231,753	(TCTTAAGTAAAGT)×2	IGS (*nad2*-exon1, *trnH*)
5	18	252,332	252,368	(CATAGTCGCGAGCTGTTT)×2	rrnL
6	15	315,763	315,793	(GTATAGTATAGTAGG)×2	IGS (*nad1*-exon1, *nad6*)
7	33	356,560	356,626	(CCTTGCCCCCTGCAGAGCCTCAAGCCCCTGA GC)×2	IGS (*atp1*, *nad4L*)

IGS: Intergenic pacers.
